# The Hapless Insane

**Published:** 1859-10

**Authors:** 


### THE HAPLESS INSANE.

Insanity may be originated in any individual; but when once originated, it may be perpetuated in the offspring for an indefinite time to come. Persons are constantly becoming insane from a great variety of causes, and yet their friends are not able to detect the slightest taint of insanity in any of their kindred.

Two facts stand out with startling import:

*First*—Of a number of idiotic children in Massachusetts, three-fourths were ascertained to have been born of parents, one or both of whom indulged freely in drinking ardent spirits.

*Second*—A gentleman of twenty-five years' experience in the management of Insane Asylums, stated on oath, in one of the courts of New York within a month or two, that three-fourths of the cases of adult insanity were clearly traceable to causes of a domestic nature. At the same time, the fortieth Annual Report of the Friends Insane Asylum, near Philadelphia, states that of all the persons admitted during 1856, the number of the unmarried insane was more than double of the married, whether as to men or women; or, to make it more clear, two-thirds of all the inmates had never been married. The contrast is certainly worthy of note, that among "Friends," a large majority of the adult insane are single persons, while among the "world's people," a still greater majority are insane from domestic causes; a result similar to this last was reached by a surgeon who inquired among soldiers as to the cause of their enlistment, "domestic difficulties" was the reply in three cases out of four.

In either case this most interesting fact presents itself, that three-fourths of all cases of unsound mind in youth and age, may be prevented by temperance as to spirituous liquors, and by well assorted marriages, and it all resolves itself into three great practical precepts:—

Get married about twenty-five.

Be loving, considerate, forbearing, wives and husbands.

Practice abstinence from all that can intoxicate until you have passed fifty years, and as much longer as a wise judgment dictates.

